# Mechanisms shared between cancer, heart failure, and targeted anti-cancer therapies

**DOI:** 10.1093/cvr/cvac132

**Published:** 2022-08-25

**Authors:** Sanne de Wit, Claire Glen, Rudolf A de Boer, Ninian N Lang

**Affiliations:** Department of Cardiology, University Medical Centre Groningen, University of Groningen, PO Box 30.001, Hanzeplein 1, 9700 RB, Groningen, The Netherlands; Institute of Cardiovascular and Medical Sciences, University of Glasgow, 126 University Place, Glasgow G12 8TA, United Kingdom; Department of Cardiology, University Medical Centre Groningen, University of Groningen, PO Box 30.001, Hanzeplein 1, 9700 RB, Groningen, The Netherlands; Institute of Cardiovascular and Medical Sciences, University of Glasgow, 126 University Place, Glasgow G12 8TA, United Kingdom

**Keywords:** Cancer, Heart failure, Cardiotoxic drugs, Mechanisms

## Abstract

Heart failure (HF) and cancer are the leading causes of death worldwide and accumulating evidence demonstrates that HF and cancer affect one another in a bidirectional way. Patients with HF are at increased risk for developing cancer, and HF is associated with accelerated tumour growth. The presence of malignancy may induce systemic metabolic, inflammatory, and microbial alterations resulting in impaired cardiac function. In addition to pathophysiologic mechanisms that are shared between cancer and HF, overlaps also exist between pathways required for normal cardiac physiology and for tumour growth. Therefore, these overlaps may also explain the increased risk for cardiotoxicity and HF as a result of targeted anti-cancer therapies. This review provides an overview of mechanisms involved in the bidirectional connection between HF and cancer, specifically focusing upon current ‘hot-topics’ in these shared mechanisms. It subsequently describes targeted anti-cancer therapies with cardiotoxic potential as a result of overlap between their anti-cancer targets and pathways required for normal cardiac function.

## Graphical Abstract

**Graphical Abstract:**
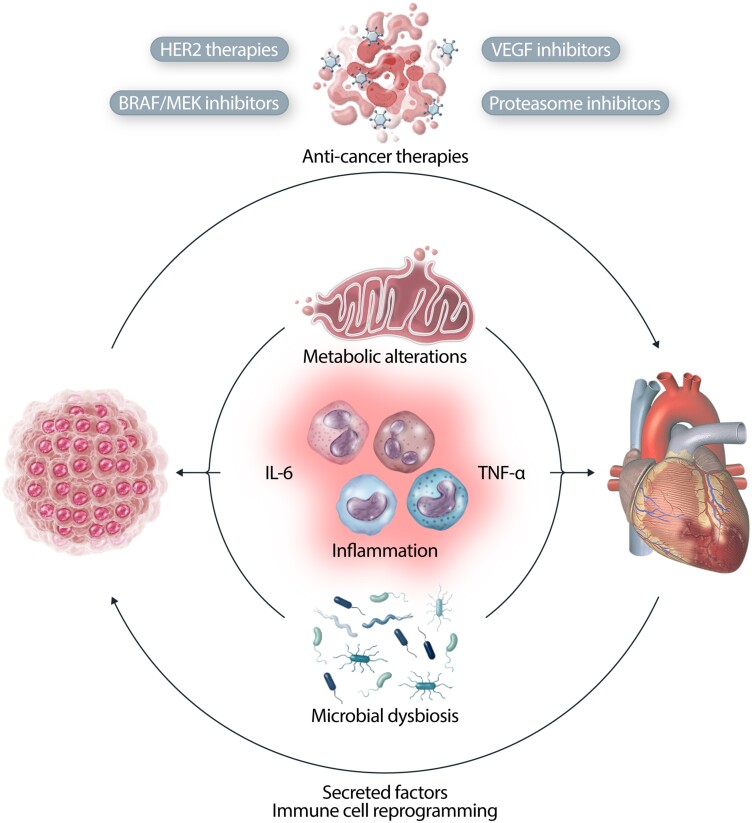

**This article is part of the Spotlight Issue on Heart Failure.**

## 1. Introduction

Cancer is the leading cause of non-cardiovascular (CV) mortality in patients with heart failure (HF),^1^ accounting for up to 9% of deaths in patients with HF with reduced ejection fraction (HFrEF) and for up to 17% of mortality in patients with HF with preserved ejection fraction (HFpEF).^2,3^ Conversely, CV disease (CVD) is the most frequent non-cancer cause of death in patients with malignancy.^4^ In addition to shared risk factors, including obesity, smoking, and diabetes, the mechanistic underpinning of the bidirectional interplay between cancer and HF is becoming clearer.^5,6^ Cancer and HF both have the potential to provoke profound alterations in cellular homeostasis. These effects are of relevance in isolation, but the substantial overlap between mechanistic pathways of tumour growth and CV physiology may also explain the increased propensity to develop the ‘other’ disease. In addition to pathophysiologic mechanisms that are shared between cancer and HF, overlaps also exist between pathways required for normal cardiac physiology and for tumour growth. Therefore, these overlaps may also explain the increased risk for cardiotoxicity and HF as a result of targeted anti-cancer therapies.

We will provide an overview of the bidirectional interactions between cancer and HF and will describe current ‘hot topic’ mechanisms shared by these conditions. We will also outline the relevance of anti-cancer therapies with potential cardiotoxic effects resulting from overlaps between their anti-cancer targets and pathways required for normal cardiac function.

## 2. Bidirectional interaction between HF and cancer

### 2.1 HF as a risk factor for cancer

Over the last decade, a growing number of clinical studies have demonstrated that patients with HF are at increased risk for developing cancer.^7–13^ The association between HF and cancer was first described in a cohort including 961 patients with HF as well as age- and sex-matched controls. Patients with HF were at increased risk of developing cancer [hazard ratio (HR): 1.68; 95% CI (1.13–2.50)], even after adjustment for body mass index, smoking, and co-morbidities.^7^ Several more recent studies have corroborated this; the most recent article to be published described a large community based study from the Puglia region of Italy (*n* = 104 020 subjects), in which cancer incidence and cancer mortality were significantly higher in patients with HF, compared to matched non-HF control subjects [HR: 1.76 (1.71–1.81) and HR: 4.11 (3.86–4.38), respectively].^13^ These findings were replicated in a German cohort, in which HF was significantly associated with the incidence of cancer [HR: 1.76 (1.71–1.81)].^11^ In the Women’s Health Initiative study, HF was associated with increased cancer incidence [HR: 1.28 (1.11–1.48)] in female patients. Notably, HFpEF but not HFrEF was associated with increased cancer incidence [HR 1.34, (1.06–1.67); and HR 0.99 (0.74–1.34), respectively].^12^

The association between HF and cancer can partially be explained by shared risk factors. However, several recent preclinical studies have shown that HF can also stimulate tumour growth directly.^14–16^ The initial evidence for a causal relation between HF and cancer comes from a study of tumour prone C57BL/6-Apc^Min^ mice, in which tumour growth increased significantly in the context of myocardial infarction (MI)-induced HF.^14^ To exclude the effect of haemodynamic impairment upon tumour growth, the experiment was repeated in a model of heterotopic heart transplantation. A higher tumour load was observed in mice with a failing heart (whether in situ or transplanted) compared to controls. Several proteins were identified to be increased in the presence of HF and were also associated with proliferative effects in colon cancer cell lines, especially Serpin3A. These observations gave rise to the hypothesis that HF may promote tumour growth through secretion of paracrine factors.^14^ This hypothesis was recently substantiated in an aortic constriction model, in which early cardiac remodelling, without severe cardiac dysfunction, was seen to promote tumour growth in a breast cancer and lung cancer mouse model.^16^ Furthermore, plasma obtained from mice subjected to aortic constriction stimulated tumour cellular proliferation, building on the evidence that secreted factors play a role in HF-induced tumour growth. Periostin was identified as a potentially important mediator, given that plasma depleted of periostin no longer evoked tumour proliferative effects.^16^ Koelwyn *et al.*^15^ demonstrated that MI-induced HF increases breast cancer growth via epigenetic remodelling of bone-marrow immune cells which resulted in an immunosuppressed, pro-cancer phenotype. However, despite multiple layers of evidence that HF can stimulate tumour growth, these effects were not reproduced in a mouse model of renal cancer, suggesting that the tumour promoting effects of HF might be cancer-site specific.^17^

### 2.2 Cancer as a risk factor for HF

The association between cancer and CVD has been demonstrated in a retrospective cohort study of over 36 000 adults surviving at least two years after a diagnosis of cancer and compared with age and sex matched non-cancer controls. This association varied by cancer type: in comparison with controls, the risk of CVD (including HF) was significantly higher in survivors of multiple myeloma [incidence rate ratio (IRR) 1.7], lung cancer (IRR 1.58) and breast cancer (IRR 1.13).^18^ However, differentiation of the effect of cancer *per se* from the cardiotoxic effects of its treatment can be difficult to disentangle in epidemiologic studies. Nonetheless, pre-clinical models show that cancer causes systemic metabolic alterations resulting in impaired cardiac function.^19,20^ Cachexia represents a systemic manifestation of both cancer and HF.^21,22^ In animal models, cancer promotes cardiac atrophy and a reduced heart weight with subsequent deterioration in cardiac function.^23,24^ Cardiac wasting appears to result from increased autophagy and myocyte apoptosis^23^ with proinflammatory cytokines including tumour necrosis factor-α (TNF-α), interleukin (IL) 1β, and IL-6 playing pathophysiological roles.^24^

## 3. Mechanistic overlap in cancer and HF pathophysiology

### 3.1 Metabolic alterations

**Figure 1:**
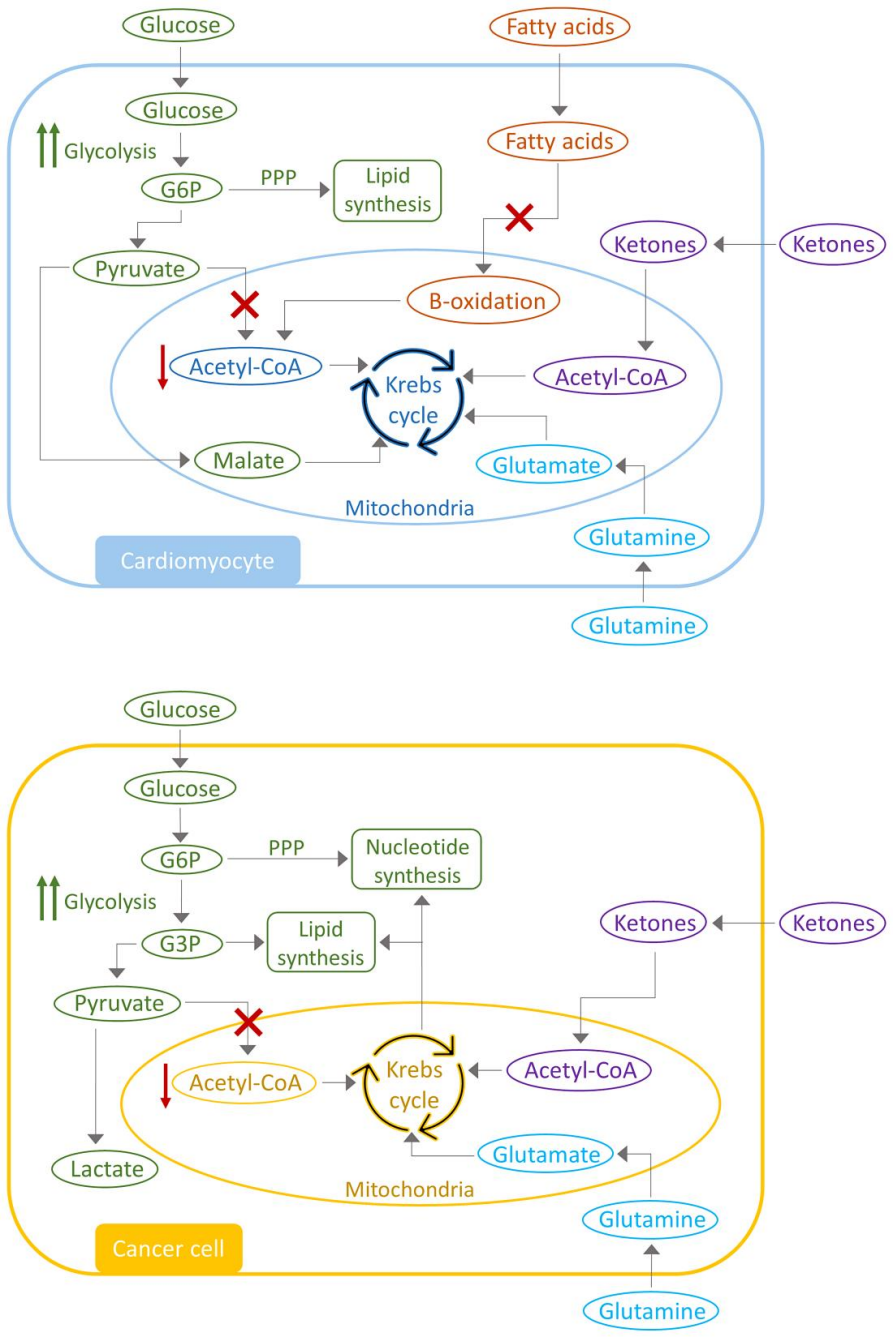
Metabolic alterations in heart failure and cancer. Heart failure and cancer are characterized by several overlapping changes in metabolic pathways. Both diseases are characterized by an increase in glycolysis and a decrease in oxidative respiration. Glycolytic intermediates are redirected into branch pathways for nucleotide and lipid synthesis. Alternative fuel sources are used for the Krebs cycle, a mechanism called anaplerosis, to compensate for the decreased acetyl-CoA levels for ATP production. Adjusted from DeBerardinis et al.^26^ and Garcia-Ropero et al.^27^

Metabolic remodelling is considered a hallmark in the pathophysiology of both cancer and HF, and has been the focus for new treatment strategies for both diseases in recent decades.^25,26^ HF and cancer are characterized by several common metabolic alterations (*Figure 1*), which begs the question as to whether metabolic derailment might play a role in the connection between HF and cancer. It remains largely unknown if metabolic switches in cancer, either in the tumour or surrounding tissues, affect the CV system. Vice versa, metabolic changes in the heart are unlikely to cause cancer development, but well-described metabolic repercussions of CVD, such as insulin insensitivity and diabetes,^28^ are clearly associated with an increased risk for cancer.

#### 3.1.1 Switch to glycolysis

One of the major commonalities in metabolic remodelling between HF and cancer is the shift in metabolic dependency, favouring glycolysis over oxidative phosphorylation. In the healthy heart, the majority of adenosine triphosphate (ATP) is produced through fatty acid (FA) oxidation and only small amounts through oxidation of glucose and ketone bodies.^29^ In the failing heart, however, glycolytic activity is increased,^30,31^ while FA and glucose oxidation is substantially decreased.^30,32^ The consequence of this is a net decrease in acetyl-coenzyme A (CoA) bioavailability for ATP production via the Krebs cycle. Similarly, cancer metabolism is also characterized by an increase in glycolytic activity. As long ago as 1927, Otto Warburg^33^ provided evidence that cancer cells obtain glucose and produce lactate irrespective of oxygen availability.^34^ Several oncogenes and tumour-suppressor genes are involved in the increased glycolysis. Specifically, phosphoinositide 3-kinases (PI3K), Ak transforming factor (Akt) and MYC proto-oncogene (MYC) are known to upregulate transcription and translocation of Glucose transporter 1, and increase hexokinase activity.^35,36^ P53, possibly the most well-known tumour suppressor gene in the field of oncology, has also been associated with metabolic remodelling, since a loss of P53 results in increased glycolytic flux.^37^

#### 3.1.2 Alternative fuel and anaplerosis

Although both cancer and HF are characterized by an increase in glycolysis, glucose is not believed to be the major energy source in either disease. In end-stage HF, ATP levels only decrease by 60–70% from normal capacity.^38,39^ In the oncology field, several studies have shown that mitochondrial metabolism is crucial for tumour proliferation^40,41^ and experimental inhibition of glycolytic ATP production, via the inhibition of pyruvate kinase, does not result in reduced tumourigenesis.^42^ These findings suggest that compensatory mechanisms are activated in HF and cancer to maintain mitochondrial ATP production.

Anaplerosis represents a mechanism via which the Krebs cycle is fuelled by intermediaries independent of acetyl-CoA. This mechanism plays an important role in both HF and cancer metabolism. Indeed, anaplerotic flux is increased in the context of cardiac hypertrophy^43,44^ and also in cancer.^45,46^ In a pressure-overload induced HF model, pyruvate was converted to malate, which can enter the Krebs cycle.^43^ Additionally, glutamine utilization is increased in HF and cancer.^47,48^ Its subsequent conversion to glutamate is followed by the production of α-ketoglutarate, an intermediate for the Krebs cycle and, even in hypoxic conditions, glutamine is utilized for oxidative ATP production in cancer cells.^49,50^ Ketone bodies are utilized as an alternative energy source in HF and this phenomenon has been reported in patients with HF^51^ as well as animal models of HF.^52^ This is believed to be an important adaptive mechanism in the setting of decreased FA oxidation. The role of ketones in cancer metabolism is less well understood but several studies have provided evidence that ketone body utilization can stimulate tumour growth,^53,54^ although data are conflicting and treatment with ketone bodies has also been reported to decrease tumour growth.^55,56^

In both cancer and HF, glycolytic intermediates are often redirected to a branching pathway for biosynthesis, instead of being directed to the Krebs cycle. In cancer, glucose-6-phosphate (G6P) is redirected to the pentose phosphate pathway (PPP) for nucleotide biosynthesis and glyceraldehyde-3-phosphate can be converted to glycerol-3-phosphate for lipid synthesis.^57,58^ In addition, Krebs intermediates are used to produce cytosolic aspartate and acetyl-CoA for nucleotide and lipid synthesis.^59,60^ In HF, G6P is also redirected to the PPP for production of nicotinamide adenine dinucleotide phosphate (NADPH), which is essential for regulating oxidative stress and lipid synthesis.^31^ These diversions of glycolytic intermediates have a negative effect upon cardiac energetics and function. Furthermore, G6P may also enter the hexosamine biosynthetic pathway, leading to increased O-GlcNAcylation, which is further associated with HF.^61,62^

#### 3.1.3 Hypoxia

Hypoxia induced factor-1 (HIF-1) is a key regulator of metabolic adaptation in cancer and HF. Proliferation of tumour cells often exceeds angiogenesis, resulting in a hypoxic environment and HIF-1 activation.^26^ Sustained activation of HIF-1 is also induced in cancer under normoxic conditions due to mutations in the mammalian target of rapamycin complex 1 (mTORC1) pathway or von Hippel-Lindau (VHL).^63^ HIF-1 regulates transcription of proteins involved in glucose metabolism and protein, lipid and nucleotide biosynthesis^64^ and has been shown to promote tumour growth.^65–67^ Interestingly, patients with VHL syndrome, characterized by sustained HIF-1 activation and development of tumours in numerous organs,^68^ also develop cardiopulmonary abnormalities.^69,70^ VHL knockout mice develop cardiac lipid accumulation, fibrosis and apoptosis.^71^ Increased HIF-1 expression also induces cardiac hypertrophy by regulating enzymes involved in FA and glucose metabolism.^72–74^ HIF-1 levels are higher in patients with hypertrophic cardiomyopathy than they are in healthy people and transverse aortic constriction (TAC) is associated with increased HIF1. Notably, ventricular depletion of HIF1α prevents TAC-induced cardiac dysfunction.^72^

#### 3.1.4 Metabolic targets in cancer and HF therapy

Because of its central role in both diseases, metabolic derangement may be a valid target in cardio-oncology. Indeed, treatments targeting metabolism in HF and cancer have been studied extensively. One promising treatment might be with sodium-glucose co-transporter 2 inhibitors (SGLT2i) which were originally developed as anti-diabetic drugs.^75^ SGLT2i drugs reduce CV events and reduce worsening HF in both diabetic and non-diabetic HF patients.^76,77^ Several preclinical studies have shown that treatment with SGLT2i can positively alter cardiac metabolism in HF models, resulting in reduced cardiac remodelling.^78–80^ The effect of SGLT2i on tumour growth has also been studied. Both *in vitro* and *in vivo*, treatment with SGLT2i inhibited tumour growth of several obesity and diabetes-associated cancer models.^81–83^ Furthermore, the SGLT2i, canagliflozin, inhibits tumour growth even in tumour models without obesity or diabetes, potentially via its effects on cellular glucose levels.^84,85^

Many studies have focused on targeting HIF-1 as an anti-cancer treatment and this has been extensively reviewed elsewhere.^86^ Indeed, HIF-1 inhibition has shown promising anti-tumourigenic effects in renal,^87,88^ hepatocellular,^89^ and breast cancers.^90^ Downregulation of HIF1 in a mouse model ischaemia/reperfusion attenuated cardiac injury after reperfusion.^91^ Interestingly, treatment with Belzutifan, an inhibitor of the HIF2 isoform, has shown promising anti-tumourigenic effects in renal cancer, was shown to attenuate pulmonary hypertension and fibrosis in mice with a VHL mutation.^92^

### 3.2 Inflammation

Inflammation has frequently been considered as a nodal point linking HF and cancer. Heart disease and cancer are associated with an increase in pro-inflammatory cytokines, including TNF-α and IL-1β^93–96^ and chronic inflammation increases the risk of new onset cancer^97,98^ and CVD.^99^ After MI, the innate immune system is activated leading to a pro-inflammatory response, which is initially cardio-protective. However, prolonged activation of pro-inflammatory signalling, especially via IL-6, induces cardiac remodelling and cardiac dysfunction.^100,101^ Importantly, pro-inflammatory cytokines also promote tumourigenesis.^102,103^ In addition, the tumour-microenvironment is infiltrated by tumour associated macrophages, which produce cytokines to stimulate angiogenesis and inhibit the anti-tumour response of cytotoxic T-cells.^104,105^

#### 3.2.1 HF-associated inflammation

Patients with HF and elevated C-reactive protein (CRP) (>2 mg/L) have an increased risk of cancer.^14,106,107^ In a pre-clinical model of MI-induced HF described previously, the subsequent effect of HF upon increased tumour growth was accompanied by elevated circulating concentrations of pro-cancer chemokines, such as chemokine (C-X-C motif) ligand (CXCL13). In these animals with HF, there was also an increase in monocytic myeloid-derived suppressor cells found in the tumour tissue and these suppressed CD8 + cytotoxic T cell activity, further potentiating tumour growth.^15^

#### 3.2.2 Clonal haematopoiesis of indeterminate potential-associated inflammation

Clonal haematopoiesis of indeterminate potential (CHIP) reflects the accumulation of somatic, potentially pro-leukemic mutations in haematopoietic stem cells, occurring in the absence of haematological malignancy.^108^ The commonest mutations found in CHIP occur in genes that are also important in the regulation of inflammation.^109^ These include mutations occurring in the driver genes DNA methyltransferase 3A (DNMT3A), ten-eleven-translocation-2 (TET2), Janus kinase 2 (Jak2) and additional sex comb-like 1 (ASXL1). Although the risk of malignant transformation is low (<1% per year), CHIP carriers have an excess risk of mortality which reflects a heightened risk for CV events including MI and stroke,^109–112^ as well as an association with increased HF hospitalization and HF mortality.^113,114^ In a large study including five population-based cohorts and over 50 000 participants, CHIP correlated with a 25% increased risk for new onset HF.^115^ Clonal haematopoiesis initiates a pro-inflammatory state associated with high circulating levels of pro-inflammatory markers in humans.^116,117^ Pre-clinical studies demonstrated that haematopoietic mutations in TET2, DMNT3A, and Jak2 lead to an accelerated HF phenotype in several mouse HF models, accompanied by an increase in pro-inflammatory cytokines, including IL-1β and IL-6.^118–120^

#### 3.2.3 Obesity-associated inflammation

Obesity is characterized by chronic inflammation. In 2016, 39% of the adult world population was overweight, of whom 14% were obese.^121^ In the lean state, adipose tissue is infiltrated by anti-inflammatory immune cells which are important regulators of insulin sensitivity.^122,123^ However, in the obese state, a shift in constituent immune cells occurs with a relative decrease in anti-inflammatory components and an increase in pro-inflammatory Th1 and CD8+ T cells. In addition, a shift occurs in the macrophage phenotype, with increased M1-like macrophages.^124^ This is accompanied by an increase in pro-inflammatory cytokines, chemokines, and adipokines, such as leptin. Numerous studies have shown that obesity increases the risk of a wide range of malignancies, including breast, colorectal, and liver cancer.^125–129^ Cytokines and adipokines secreted by adipose tissue stimulate tumour growth and progression, including IL-6, TNFα, and leptin.^130,131^ In addition, cancer-associated adipocytes may also be present in the tumour-microenvironment and can further stimulate tumour progression.^132^ Obesity is also associated with and increased risk of HF, especially HFpEF^133–136^ and it is notable that leptin-resistant db/db mice develop a HFpEF phenotype, with evidence of cardiac hypertrophy and interstitial fibrosis.^137^ IL-6, TNFα, and leptin also induce hypertension and atherosclerosis,^138,139^ both of which represent major pathogenetic processes in the development of HF.

#### 3.2.4 Inflammation as a therapeutic target in HF and cancer

Considering the role of inflammation in both diseases, targeting inflammatory mechanisms in HF and cancer could have therapeutic potential. Indeed, commonly used HF medications, such as statins, have some anti-inflammatory properties.^140^ In the Canakinumab Anti-Inflammatory Thrombosis Outcome Study (CANTOS), treatment with canakinumab, a monoclonal IL-1β antibody, reduced the rate of recurrent atherosclerotic CV events in patients with previous MI and high CRP levels (<2 mg/L).^141^ Notably, canakinumab also reduced HF hospitalization and HF-related mortality by 23% in patients who achieved a CRP level of <2 mg/L.^142^ A sub-analysis of the CANTOS study showed that treatment with Canakinumab also decreased the incidence of lung cancer^143^ and is being investigated further as a treatment for that indication.^144^

Targeting inflammatory chemokines as a therapeutic strategy for HF and cancer has also generated interest. Chemokines including Chemokine (C-C Motif) Ligand 2 (CCL2) and CXCL13 play a pivotal role in cancer^145,146^ and circulating levels are also increased in patients^147,148^ and preclinical models of HF.^149^ Preclinical studies using CCL2 or CXCL13 inhibitors have reported treatment-related reductions in tumour proliferation.^150–153^ CCL2 knockout attenuates cardiac remodelling after ischaemia/reperfusion injury, but CCL2 knockout was also associated with delayed replacement of injured cardiomyocytes with connective tissue, a process that is essential after infarction.^149^ Initial clinical trials examining the use of chemokine inhibitors in cancer have shown promise.^154^ However, clinical studies in patients with HF have not yielded positive results so far.^155^ A better understanding of the complex role of chemokines in the pathophysiology of cancer and HF is required in order to maximize the potential of this potential strategy.

The complex inter-relationship between the immune system, cancer and HF is exemplified by immune checkpoint inhibitors in use as anti-cancer therapy. These potent anti-cancer drugs now have a very broad, and growing, range of indications in oncology and are associated with remarkable cancer outcomes.^156,157^ By inhibiting immune checkpoints on cancers cells, a T cell mediated immune response is initiated, allowing immune targeting of cancer cells.^156^ However, myocarditis occurs in up to 2% of patients treated with these agents and CV mortality associated with these events can be up to 40%.^158–160^

### 3.3 Microbiome

**Figure 2:**
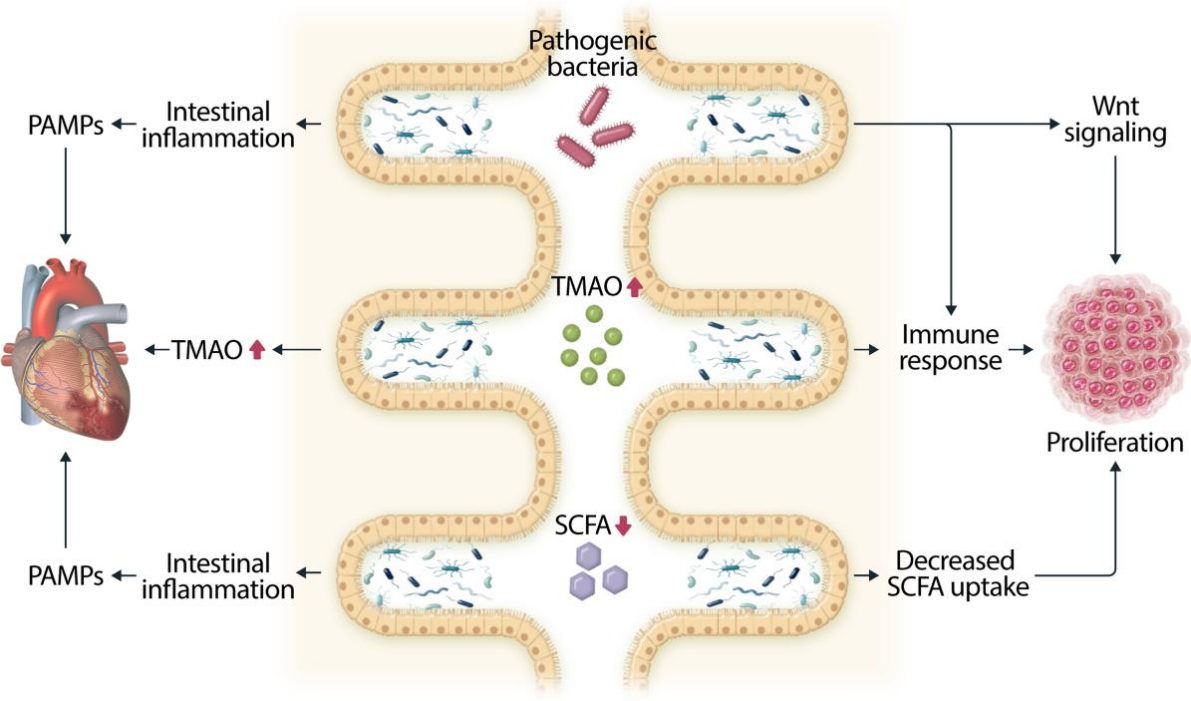
The overlapping effects of microbial dysbiosis in HF and cancer. Overgrowth of pathogenic bacteria and decreased SCFA levels can induce intestinal inflammation, resulting in increased circulating PAMPs, which can induce cardiac fibrosis and dysfunction. Increased circulating levels of microbial metabolite TMAO can induce atherosclerotic plaque formation and HF. In cancer increased TMAO and pathogenic bacteria can directly promote tumour growth by activating immune cells. In addition, pathogenic bacteria can directly promote tumourigenesis by activating Wnt/β-catenin signalling. Decreased SCFA levels are favourable for tumourigenesis and tumour cells actively inhibit SCFA uptake.

The human microbiome is comprised of trillions of bacteria, archaea, and eukaryotic microbes, which maintain a mutualistic relationship with their host.^161,162^ Gut microbiota are essential for fermentation of dietary fibres and vitamin biosynthesis, and play an important role in intestinal health and immune regulation.^161,163^ The microbial composition is greatly affected by environmental factors such as food and dietary patterns, smoking and drug use. These factors can influence the microbial diversity and the abundance of specific microbial species, resulting in microbial dysbiosis.^164^ People with obesity, for instance, show decreased microbial diversity.^165^ Accumulating evidence is emerging on the bidirectional connection between the microbiome, HF and cancer. HF and cancer, and their therapies, are believed to affect the microbial composition in several ways and microbial dysbiosis can play a role in both diseases as outlined below and in *Figure 2*.

#### 3.3.1 Microbial dysbiosis in cancer and HF

The role of the gut microbiome in colorectal cancer (CRC) has been studied extensively. Patients with CRC exhibit a distinct microbial composition, characterized by an increase in *Fusobacteria* and pathogenic bacteria and a decrease in *Clostridiales*, *Faecalibacterium*, and *Bifidobacterium.*^166–168^ In addition, microbial dysbiosis has been associated with an increased incidence of CRC.^166,167^  *In vivo* studies have provided direct evidence that microbiota from patients with CRC can promote tumourigenesis.^169,170^ In these studies, stool samples from patients with CRC were transplanted into a colon cancer mouse model, or germ-free mice. Stool samples from patients had different microbial composition than stool samples from healthy individuals, and transplantation of microbiota from CRC patients led to increased tumour growth in the colon cancer model and increased colonocyte proliferation in the germ-free mice.^169,170^ Microbial dysbiosis has also been associated with cancer in other organs, including breast and liver.^171,172^

Substantial clinical evidence now exists to show that patients with HF also have a distinct gut microbial composition.^173–175^ The microbiome and HF appear to affect one another in a bidirectional way. Differences in microbial composition can increase atherosclerotic plaque formation and can lead to a worsening HF phenotype in mouse models.^176–178^ On the other hand, HF may cause gut congestion and low-grade inflammation, resulting in increased intestinal permeability, and consequent microbial dysbiosis.^179,180^ In addition, HF medication plays a role in the equation. HF medication can strongly affect the microbial composition ^181,182^ and vice versa, the microbiome can affect the response on HF drugs, altering the effectiveness of treatment.^183^

Notably, there are several similarities in between microbial patterns observed in cancer and those observed in HF, including an increase in *Fusobacteria* and a decrease in *Clostridiales*, and *Bifidobacterium*. Furthermore, the microbiome has been shown to influence both HF and cancer via mechanisms including pathogenic bacteria, microbial metabolites and short chain FAs (SCFAs) (*Figure 2*).

#### 3.2.2 Pathogenic bacteria

Several bacteria have been identified to have direct tumourigenic effects, including *Helicobacter pylori*, *Fusobacterium nucleatum*, *Bacteroides fragilis*, and *Salmonella enterica*. *H. pylori* bind to intestinal epithelial cells and interact with Wnt/β-catenin signalling, directly regulating cell proliferation, inflammation and apoptosis.^184^  *F. nucleatum* has the ability to induce tumour proliferation by inducing a proinflammatory response through activation of the nuclear factor-κB (NF-κB) pathway^185^ and is associated with epithelial-to mesenchymal transition.^186^  *B. fragilis* targets intestinal tight junctions through activation of the Wnt/β-catenin and Nf-kB pathways, resulting in barrier disruption and intestinal inflammation^187^ and can induce proliferation through activation of celullar MYC (C-MYC).^188,189^

Several pathogenic bacteria are more abundant in patients with HF. Pathogenic bacteria affect gut barrier integrity, resulting in bacterial translocation and chronic inflammation, which has been associated with CVD.^190^ Indeed, systemic levels of several pathogen-associated molecular patterns (PAMPs), such as lipopolysaccharide (LPS), are increased in patients with HF.^191^ LPS exposure leads to cardiac fibrosis and dysfunction in mice.^192,193^

#### 3.3.3 Trimethylamine-N-oxide

Arguably the best characterized link between HF and the microbiome is that of trimethylamine-N-oxide (TMAO).^194,195^ This microbial metabolite is generated from nutrients containing trimethylamine (TMA), including choline and l-carnitine. TMA is transported to the liver where it is further metabolized to TMAO. Circulating TMAO levels are associated with an increased risk for CVD and HF, and worse outcomes.^196–199^ These observations have been corroborated in preclinical studies, which provide evidence of direct effects of TMAO on atherosclerotic plaque formation and HF.^176–178^

There is increasing evidence to suggest that TMAO also plays a role in tumour growth. Higher TMAO levels have been associated with an increased risk for CRC in several studies.^200–202^ However, not all investigators have demonstrated this link in CRC.^203^ It is of note that any association between and TMAO and cancer may extend beyond the gastrointestinal tract and higher TMAO levels have also been associated with prostate cancer and oral squamous cell carcinoma.^204,205^ It is currently hypothesized that TMAO can induce tumourigenic effects by activating a chronic immune response.^206,207^

#### 3.3.4 SCFAs

A key signature in the microbial composition found in patients with CRC as well as patients with HF is the substantially lower number of SCFA-producing bacteria, such as *Roseburia and Lachnospiraceae.*^168,175,208,209^ SCFAs, including acetate, propionate and butyrate, are fermentation products of dietary fibres, produced by gut microbiota. Butyrate is the main energy source for colonocytes and is essential for maintaining colonic health, mucus production, barrier integrity and immune regulation.^210^ However, cancer cells switch energy source, favouring glucose over butyrate,^211^ and butyrate uptake in colon cancer cells is substantially decreased.^212,213^ Furthermore, several preclinical studies provide evidence that butyrate possesses anti-tumourigenic properties and that butyrate treatment inhibits tumour growth in CRC cell lines.^214,215^

Low SCFA levels are also associated with hypertension and HF. Decreased SCFA levels can cause intestinal barrier dysfunction, resulting in translocation of microbial metabolites as well as inflammation.^216^ Plasma SCFA levels are decreased in patients with HF.^217^ and SCFA supplementation attenuates HF in mice.^218–220^ Butyrate supplementation protected from doxorubicin induced cardiotoxicity *in vivo*.^218^ Propionate induces vasodilatation and antibiotic-induced depletion of propionate is associated with increased blood pressure in mice.^221^ Whether or not this afterload effect is a relevant mechanism for the development or worsening of HF in humans is unknown.

## 4. Mechanistic overlap in anti-cancer targeted therapies and cardiac physiologic pathways

In addition to shared pathophysiological mechanisms in cancer and HF, there are also substantial overlaps between pathways required for tumour growth and those required for normal cardiac function. These overlapping effects make substantial contributions to the potential cardiotoxic effects of a growing number of anti-cancer therapies, with consequent left ventricular dysfunction and HF. In contrast to traditional chemotherapeutic agents, the majority of novel anti-cancer therapies are targeted and act upon specific cancer signalling pathways. Numerous targeted therapies have been associated with HF, including anti-human epidermal growth factor receptor 2 (HER2) therapies, vascular endothelial growth factor (VEGF) signalling pathway inhibitors, rapidly accelerate fibrosarcoma B-type (BRAF), and mitogen-activated extracellular signal-regulated kinase (MEK) inhibitors and proteasome inhibitors (*Table 1*).

**Table 1:** Mechanisms of cardiotoxicity due to targeted therapies

Targeted therapy class	Examples	Overlapping signalling pathways for cancer growth and cardiac physiology	Proposed mechanisms of cardiotoxicity
HER2 + targeted therapy	Trastuzumab Pertuzumab	HER2 NRG-1 HER4 PI3K/Akt MEK/ERK Src/FAK mTOR	Impaired cell proliferation
			Impaired angiogenesis
			Impaired cardiomyocyte metabolism
			Impaired autophagy
			Impaired mitochondrial function
VEGF inhibitors	Axitinib Cabozantinib Lenvatinib Pazopanib Sunitinib Sorafenib Vandetanib	RAF/MEK/ERK PI3K/Akt VEGFR PDGFR c-Kit AMPK	Hypertension
			Capillary rarefaction
			Cardiomyocyte apoptosis
			Oxidative stress
BRAF/MEK inhibitors	Dabrafenib + Trametinib Encorafenib + Binimetinib Vemurafenib + Cobimetinib	RAF/MEK/ERK	Hypertension
			Cardiomyocyte apoptosis
			Impaired cardiomyocyte hypertrophy
Proteasome inhibitors	Bortezomib Carfilzomib Ixazomib	Ubiquitin-proteasome system	Altered protein homeostasis
			Accumulation of misfolded proteins
			Endothelial dysfunction
			Cardiomyocyte apoptosis

HER4, human epidermal growth factor receptor 4; RAF, rapidly accelerate fibrosarcoma.

### 4.1 Anti-HER2-targeted therapies

The transmembrane tyrosine kinase receptor HER2 is overexpressed in approximately 20% of breast cancers.^222^ Trastuzumab is a monoclonal antibody against HER2 and improves survival in patients with HER2 positive breast cancer. However, it is strongly associated with cardiac toxicity. A meta-analysis of ten randomized controlled trials of trastuzumab reported that, during trial follow-up, asymptomatic left ventricular systolic dysfunction (LVSD) and symptomatic HF occurred in 7.5 and 1.9% respectively.^223^ Registry data from the United States of America reveal that the incidence of HF is up to 6% at 1 year and 20% at 5 years.^224^

HER2 and its ligand, neuregulin-1 (NRG-1), are essential for embryonic heart development, cardiomyocyte growth and survival and maintaining cardiac function in the adult heart.^225,226^ Cardiac-specific HER2 murine knock-out animals are apparently normal at birth but develop dilated cardiomyopathy as they age.^226,227^ In the physiologic state, homo- or hetero-dimerization of HER2 activates downstream signalling pathways including the PI3K/Akt which protects cardiomyocytes from apoptosis and also activates MEK/extracellular signal-regulated kinase (ERK) pathways which promote cardiomyocyte growth and proliferation.^228^

Trastuzumab binds to the extracellular segment of HER2 receptors and blocks downstream PI3K/Akt activity while pertuzumab, a more recently introduced anti-HER2 monoclonal antibody, inhibits signalling via inhibition of dimerization.^229^ Inhibition of HER2 results in a downregulation of endothelial nitric oxide synthase (eNOS) expression, accumulation of reactive oxygen species (ROS) and subsequent acceleration of apoptosis causing oxidative stress and cell injury.^230^ Furthermore, inhibition of HER2 downregulates the MEK/ERK and Src/focal adhesion kinase (FAK) pathways resulting in disordered myocardial structure.^231^ Recent studies have also shown that inhibition of HER2 with trastuzumab, in human primary cardiomyocytes, activates the Erk/mechanistic target of rapamycin (mTOR) cascade which leads to autophagy inhibition, ROS accumulation and reduced mitochondrial function.^232^

### 4.2 VEGF signalling pathway inhibitors

Angiogenesis, the process of new blood vessel growth, is vital for the nutrient supply and growth of solid organ cancers. VEGF is the most potent angiogenic factor and VEGF signalling pathway inhibitors (VSPIs) are effective anti-cancer therapies used in the treatment of a wide range of cancers including renal, hepatocellular and thyroid cancers, gastrointestinal stromal tumours, sarcoma and others. Numerous VSPIs have been developed including monoclonal antibodies and tyrosine kinase inhibitors (TKIs). Hypertension is the most commonly described CV adverse effect associated with VSPIs^233,234^ but these drugs are also associated with LVSD and HF.^235^ Meta-analysis of 21 trials including several VSPIs reported an incidence of LVSD of 2.4%.^235^ In addition to a fundamental role in the control of angiogenesis, VEGF signalling plays a pivotal role in endothelial cell proliferation and survival^236^ and acts as a compensatory mechanism in response to cardiac stressors. VEGF is secreted in response to hypertension and ischaemia and plays a key role in cardiomyocyte hypertrophy.^237,238^

Mechanisms of cardiotoxicity and HF related to VEGF inhibitors are thought to result from a combination of direct myocardial toxicity due to a reduction of cardioprotective signalling and increased cardiac afterload. The inhibition of VEGF signalling in animal models of pressure overload leads to capillary rarefaction, reduced contractile function and the development of HF.^239^ Preliminary data suggest a role for cardiac microvascular dysfunction in the development of LVSD in patients treated with VSPI.^240^ Additionally, VEGF TKIs also act on a range of non-VEGF targets and this varies between drugs. While sometimes intended to increase anti-cancer effects, these non-VEGF target effects may also be unintended or incompletely defined. Potentially cardiotoxic non-VEGF or ‘off-target’ effects include inhibition of platelet derived growth factor receptor (PDGFR) or adenosine monophosphate kinase (AMPK) downregulation.^241,242^

### 4.3 BRAF and MEK inhibitors

BRAF and MEK are key components of the mitogen activated protein kinase (MAPK) pathway, a key regulator of normal cell growth and proliferation. Mutation of the BRAF gene results in constitutive activation of BRAF’s kinase function and may be found in patients with melanoma,^243^ non-small cell lung cancer^244^ and CRC.^245^ The use of these drugs has had a profound impact on outcomes for patients with melanoma in particular. Inhibition of BRAF alone is associated with drug-resistance due to paradoxical hyperactivation of MEK, and combined inhibition of BRAF and MEK helps minimize resistance and improve outcomes.^246^ Treatment with BRAF and MEK inhibitors in combination is associated with an increased risk of HF compared to BRAF inhibitor monotherapy. Meta-analysis of five randomized controlled trials reported reduction in LVEF in 8.1% of patients in the combination therapy group compared to 2% in the BRAF inhibitor monotherapy group.^247^

Activation of MAPK signalling leads to a cascade of phosphorylation events and ultimately the activation of ERK. Activated ERK provokes the phosphorylation of several targets involved in the regulation of key cellular activities.^248^ As such, the MAPK pathway is a key component in processes including myocyte hypertrophy, cardiac remodelling and myocardial cell death.^249^ Animal models have also demonstrated that the MEK/ERK signalling pathway is required for the protection of myocardium following ischaemic injury.^250^ Disruption of the MAPK pathway by BRAF and MEK inhibitors could therefore lead to a change in these physiological cardioprotective mechanisms and affect apoptosis, remodelling and hypertrophy, ultimately leading to LVSD and HF.^249,251^ In mouse models, ERK null mice have normal cardiac function but are more susceptible to a subsequent cardiac insult.^250^ Therefore, a ‘second hit’ such as hypertension or ischaemia be the trigger to LVSD in the context of BRAF and MEK inhibitor exposure.^252^

### 4.4 Proteasome inhibitors

The proteasome is a protein complex which plays an important role in intracellular protein degradation and influences a number of intracellular processes.^253^ Proteasome inhibition leads to an accumulation of misfolded intracellular proteins, an unfolded protein stress response, with subsequent cell-cycle arrest and apoptosis which is toxic to cancer cells. Proteasome inhibitors including bortezomib, carfilzomib and ixazomib are used in the treatment of haematological malignancies including multiple myeloma and certain lymphomas.^254^ Meta-analysis of 25 clinical trials showed that bortezomib did not significantly increase the risk of cardiotoxicity compared to control patients.^255^ However, meta-analysis of 24 clinical trials of carfilzomib reported an incidence of HF of 4.1%^256^ which may be a reflection of carfilzomib’s irreversible action.

The ubiquitin-proteasome system is essential for the turnover of damaged or misfolded proteins to balance the synthesis of new proteins in the heart.^257^ Specific proteins are labelled with ubiquitin molecules identifying them for degradation by the proteasome. Optimal cardiomyocyte function is dependent on this equilibrium between protein synthesis and turnover. Patients with advanced HF and hypertrophic cardiomyopathy have reduced myocardial proteasome activity with a resulting relative increase in the ratio between protein synthesis and degradation.^258^ Bortezomib reversibly inhibits and carfilzomib irreversibly inhibits the 26S proteasome.^254^  *In vitro* studies have shown that both bortezomib and carfilzomib are directly toxic to cardiomyocytes and induce apoptosis.^259^ Mouse models with genetically modified ubiquitin-proteasome activity and pressure overload showed a marked increase in cardiomyocyte death causing rapidly progressive HF.^260^ A recent study demonstrated that dysregulation of the ubiquitin-proteasome system and expression of truncated titin proteins is implicated in the pathogenesis of dilated cardiomyopathy associated with truncating variants in the TTN gene.^261^ Engineered muscle generated from human induced pluripotent stem cell-derived cardiomyocytes with truncating variants in TTN showed an improvement in function in response to proteasome inhibition.^261^ This is in contrast to the effects of proteasome inhibition seen in the context of cancer therapy and further work is welcomed to enhance knowledge in this area.

## 5. Conclusion

The bidirectional interplay between cancer and HF is substantial and relates to several fundamental mechanisms. It now remains to be seen whether therapeutic targeting of these common pathophysiologic pathways can be harnessed to allow improved outcomes for patients affected by HF or cancer or, indeed, in the growing population affected by both. Furthermore, the adverse cardiac effects of a growing number of targeted anti-cancer therapies have, inadvertently, provided insight into the relevance of pathways required for normal cardiac function. These overlaps serve to further reinforce the growing relevance and need for close collaboration between cancer and CV specialists, in clinical, basic science, and drug development settings.
